# Fertility and reproductive outcome after tubal ectopic pregnancy: comparison among methotrexate, surgery and expectant management

**DOI:** 10.1007/s00404-020-05749-2

**Published:** 2020-08-27

**Authors:** Silvia Baggio, Simone Garzon, Anna Russo, Cesare Quintino Ianniciello, Lorenza Santi, Antonio Simone Laganà, Ricciarda Raffaelli, Massimo Franchi

**Affiliations:** 1 Department of Obstetrics and Gynecology, AOUI Verona, University of Verona, Verona, Italy; 2 Department of Obstetrics and Gynecology, “Sacro Cuore” Hospital, Negrar Di Valpolicella, Verona, Italy; 3 Department of Obstetrics and Gynecology, “Filippo Del Ponte” Hospital, University of Insubria, Varese, Italy; 4 Department of Endocrinology, Diabetes and Metabolism, AOUI Verona, University of Verona, Verona, Italy

**Keywords:** Ectopic pregnancy, Methotrexate, Fertility, Salpingectomy, Pregnancy outcome

## Abstract

**Purpose:**

To compare fertility and reproductive outcome after surgical, medical, and expectant management for tubal ectopic pregnancy (EP).

**Methods:**

133 of 228 patients, who were managed between January 2012 and December 2017 for a tubal EP, tried to conceive immediately after treatment: 86 out of 173 (49.7%) underwent surgical treatment; 38 (21.9%) were treated with methotrexate (MTX), and 49 (28.3%) had expectant management. Clinical data were retrieved by medical records, fertility outcomes were obtained by phone follow-up. The cumulative incidence (CI) of intrauterine clinical pregnancy (CP), miscarriage, live birth (LB), and recurrent EP, and the time between treatment and first intrauterine CP were compared between women treated with MTX, surgery and expectant management.

**Results:**

The CI of intrauterine CP starting from 12 months after the EP was 65.3% for the expectant management, 55.3% for the MTX group, and 39.5% for surgery (*p* = 0.012). Post-hoc analysis showed expectant management having higher intrauterine CP and LB, and shorter time between treatment and first intrauterine CP compared to surgery (*p* < 0.05). The CI of recurrent EP was comparable between the 3 groups. The analysis stratified per βhCG cut-off of 1745 mUI/mL and EP mass cut-off of 25 mm reported consistent results.

**Conclusions:**

Women successfully managed by expectation appear to have better reproductive outcomes compared to women who underwent surgery, with the shortest time to achieve a subsequent intrauterine CP. Therefore, if safely applicable the expectant management should be considered in the case of tubal EP. The fact that the chosen treatment was primarily guided by the βhCG value and EP mass diameter based on the protocol, which is intrinsically related to the characteristics of the EP, represents the main limitation of the present study. Indeed, we cannot completely exclude that the observed differences between treatments are related to the EP itself instead of the treatment.

## Introduction

Ectopic pregnancy (EP) occurs when the fertilized ovum implants outside the endometrial cavity with an incidence of 1% of all pregnancies [1]. Tubal localization accounts for 95–99% of ectopic pregnancies. Other localizations such as ovarian, cervical, cornual, and abdominal implants are rarely seen [2, 3]. In the past decades, the management of EP was revolutionized by the development and continuous improvement of transvaginal ultrasonography (TVUS), which, together with the implementation of the Beta human chorionic gonadotropin (βhCG) assay, allows early diagnosis of EP with the prevention of complications [4]. As a consequence, the clinical presentation of EP has changed from a life-threatening disease, necessitating emergency surgery, to a benign condition in almost asymptomatic women for whom non-surgical treatment options are also available [5].

With the development of laparoscopic techniques, rapid and mini-invasive intervention is quite always possible [6], which may be radical, by the removal of the entire fallopian tube (salpingectomy), or conservative, with the only removal of the products of gestation from the tube by salpingotomy or tubal milking [7, 8]. Non-surgical treatments of EP include expectant management and intramuscular or intravenous Methotrexate (MTX) injection, which avoid the potential complications of surgery. Treatment of uncomplicated EPs with MTX was reported effective, safe, and less costly as compared to surgery [5, 9].

The best approach is tailored to the patients’ medical status, success rate, complications rate, side effects, and costs. Nevertheless, even fertility outcomes after the episode are important variables to consider in choosing the treatment modality [10]. However, it remains unclear which treatment is the best regarding subsequent fertility [11], in particular, there are still insufficient data about treatment success and future fertility rates in EP cases managed expectantly [12–15]. On that bases, we performed a cohort study aimed to compare the success rate and the impact on fertility and reproductive outcomes of the surgical, medical, and expectant management for tubal EP.

## Materials and methods

### Study population

Women diagnosed with an EP between January 2012 and December 2017 in the department of Obstetrics and Gynecology of the “Ospedale Donna Bambino” in Verona (Italy) were identified searching in the records of all hospitalization performed during that period, using the diagnosis-related group (DRG) of EP at admission and/or at discharge. We also performed a crossmatch search among Gynecological Department DRG data and the Pharmacological Department Registers, to identify all gynecological patients who received an injection of MTX. Moreover, a crossmatch search was performed with the Gynecological Surgical register to identify the records of all surgeries performed for EP. The medical records of all identified cases were retrieved, and the data were extracted.

Patients who were admitted for the first episode of clinically suspected tubal EP were eligible. When one patient had more than one EP during the study period, only the first episode was considered. If the patient had the first episode out of the study period or at another center, the woman was excluded from the study. Patients with no tubal EP (cervical, ovarian, cornual, subhepatic, interstitial implantation) or EP with unknown localization were excluded. Clinically suspected EP was defined by the presence in the medical records of two criteria: direct transvaginal ultrasound (US) showing specific signs, such as hematosalpinx or lateral uterine gestational sac and empty uterus; and positive βhCG having suggestive kinetics, such as no doubling, little reduction or stability of βhCG levels after 48 h. We considered eligible only patients with an empty uterus and a sonographic suspect of tubal EP mass, in order to exclude early spontaneous miscarriages. The dimension of the gestational sac was retrieved by medical records; if a gestational sac was not reported, the patient was excluded from the current analysis. The presence or absence of clinical signs or symptoms, such as pelvic pain and metrorrhagia, were not required.

For all eligible patients, a phone follow-up was performed after 12–80 months from treatment, only one telephone call for each patient was done. Women reporting no desire of subsequent pregnancy after the index EP, and who, therefore, did not actively try to conceive were excluded. Patients who actively tried to conceive after the index EP and who accepted to be involved in the study were included. After consent and inclusion, a telephone interview was submitted with questions focused on fertility outcomes after the index EP. The questions were aimed to investigate the diagnosis of intrauterine clinical pregnancy (CP), miscarriages (pregnancy loss before 24 gestational weeks), live births (LBs) (delivery after 24 gestational weeks), recurrent EP, mode of conception, and details about any subsequent surgery after the index EP. The time between the treatment of the index EP and each recorded outcome was estimated from the date of the index EP treatment (or discharge in case of expectant management) until the last period date before the CP or recurrence EP.

Demographic characteristics of included patients such as age, smoking status, body mass index (BMI), gravid, parity before index EP (miscarriage, abortion, previous EP, live births), blood group, and Rh status were retrieved from medical records. From medical records, data regarding the access at the emergency department on the day of diagnosis (cause of the access, symptoms, levels of βhCG, hemoglobin, platelets, white cells, AST, ALT, Creatinine, and US main diameter of the mass indicated in the report) were also retrieved. All data were collected in a database.

### Management

According to the protocol adopted at the “Ospedale Donna e Bambino” and consistent with the literature (Table 1) [4, 11], after sonographic, biochemical, and clinical evaluation, the gynecologist decided the treatment of the clinically suspected tubal EP. Those who did not require an immediate surgical intervention underwent repeated blood tests for βhCG levels and complete blood cell count, as well as repeated US examinations and measurements of blood pressure and pulse, in order to decide the best treatment, according to the protocol.

**Table 1:** Management protocol of ectopic pregnancy applied to the study population

Admission	EP mass diameter	Evaluation of βhCG after 48 h		
mUI/mL		**↑**	**≈**	**↓**
βhCG ≤ 1000	< 35 mm	MTX	Expectant/MTX	Expectant/MTX
	Absent	Expectant	Expectant	Expectant
1000 < βhCG ≤ 2000	< 35 mm	Surgery	Surgery/MTX	MTX/expectant
	Absent	Monitoring + curettage/MTX	Monitoring + curettage/(MTX)	Expectant/MTX
βhCG > 2000	< 35 mm	Surgery	Surgery	Surgery/MTX
	Absent	Surgery/MTX	Surgery/MTX	Expectant + curettage/MTX

*EP* ectopic mass, *βhCG* beta-human chorionic gonadotropin, *MTX* methotrexate

In the case of expectant management or medical treatment, women were discharged and followed up in the outpatient regimen. They must be asymptomatic during the hospitalization and they had to check βhCG values every 7 days until negative values (< 5 UI/L). In the case of pain or rising of the βhCG levels, the management option was reconsidered.

Patients were eligible for medical treatment consistently with protocol showed in Table 1 and if they satisfied the following conditions: (a) no intrauterine pregnancy sac on US (synchronous orthotopic pregnancy); (b) hemodynamic stability; (c) normal results of the liver and renal function tests; (d) patient’s consent; and (e) no known allergy to MTX. Medical treatment consisted of a single dose of intramuscular injection of MTX (50 mg/m^2^), without the alternating administration of folinic acid [16]. The protocol required that the βhCG levels were measured on days 0, 4, and 7 after MTX. The success of medical treatment was defined as the drop of βhCG levels between days 4 and 7, which allowed for weekly biological follow up till resolution (βhCG < 5 UI/L). Conversely, a reiteration of medical management with reinjection of MTX (50 mg/m^2^) was required when there was a growth of βhCG levels between days 4 and 7 or if during serial biological monitoring βhCG value remained ≥ 5 UI/L. Failure of medical treatment was considered when the growth of βhCG levels was observed despite the repetition of MTX injection or patient clinical worsening after the first or second MTX injection. Those receiving MTX were informed about the possible side effects and the interactions with alcohol, non-steroidal anti-inflammatory drugs, aspirin, and antibiotics, and received advice on fluid intake, buccal hygiene, and exposure to sunlight. They were advised to use adequate contraception for 3 months after the last injection of MTX [17].

In no symptomatic women with EP mass < 35 mm and free fluid in Douglas lower than 100 mL, both expectant management and MTX treatment were possible. The decision between the two managements was made considering mainly βhCG levels and its dynamic after 48 h. Unless rapid increase of βhCG, expectant management was preferred for values < 1000 mUI/mL or in case of decreasing trend; for values stable between 1000 and 2000 mUI/mL, both expectant and MTX were considered, although MTX management was usually preferred (Table 1).

The surgical approach was offered to patients with severe symptoms or signs suggesting surgical complications (intra-abdominal bleeding > 100 mL, acute abdomen, shock, presence of the extrauterine fetal heartbeat, diameter of the mass > 35 mm), these patients were immediately treated surgically. Moreover, surgical treatment was performed in case of high stable or increasing βhCG values or if previous treatments failed. There were three options for surgical treatment: tubal or fimbriae milking, salpingotomy, and salpingectomy. Salpingectomy was the treatment of choice at our Institution, due to the reported lower rate of recurrence with no significant difference in future fertility compared to salpingotomy [7, 18, 19]. To evaluate fertility outcomes, we considered only the final treatment.

### Statistical analysis

Statistical analysis was performed using SPSS for Windows V.21.0 (IBM Corporation, Armonk, NY). Kolmogorov–Smirnov test was used to determine if continuous variables were normally distributed. Descriptive statistics were reported according to data distribution as mean ± standard deviation (SD), or median and range for continuous variables; the nominal variables were reported as absolute number and percentage (%). Normally distributed variables were compared between the three groups using the ANOVA test and post-hoc analysis as appropriate. Kruskal–Wallis test by ranks was used to compare nonparametric continuous and ordinal variables with post-hoc analysis if required. Qualitative data were compared using the Chi-square test or Fisher’s exact test when the expected frequency was less than five. Statistical significance was set at *p* < 0.05, after adaptation in case of multiple comparisons with the Bonferroni method. To evaluate whether the βhCG and the EP mass diameter were possible confounders regarding the fertility outcomes, we repeated the analysis between the three groups strafing the study population based on the βhCG cut-off value of 1745 mIU/mL, previously reported associated to tubal patency [20], and based on the EP mass diameter of 25 mm.

## Results

In our study period, 228 women were admitted at the University Hospital of Verona with a diagnosis of clinically suspected EP. Twelve of 228 (5.2%) were excluded from the study population due to extra tubal location of the ectopic pregnancy, in particular 2 (0.9%) had ovarian, 3 (1.3%) cervical, 1 (0.4%) interstitial, 1 (0.4%) subhepatic [21], 1 (0.4%) uterine isthmic and 4 (1.7%) cornual locations. Twelve of 228 (5.2%) women had a history of tubal EP but were excluded from the study because the previous hospitalization was elsewhere and information was not available, or because the previous EP was not during the study period.

**Fig. 1:**
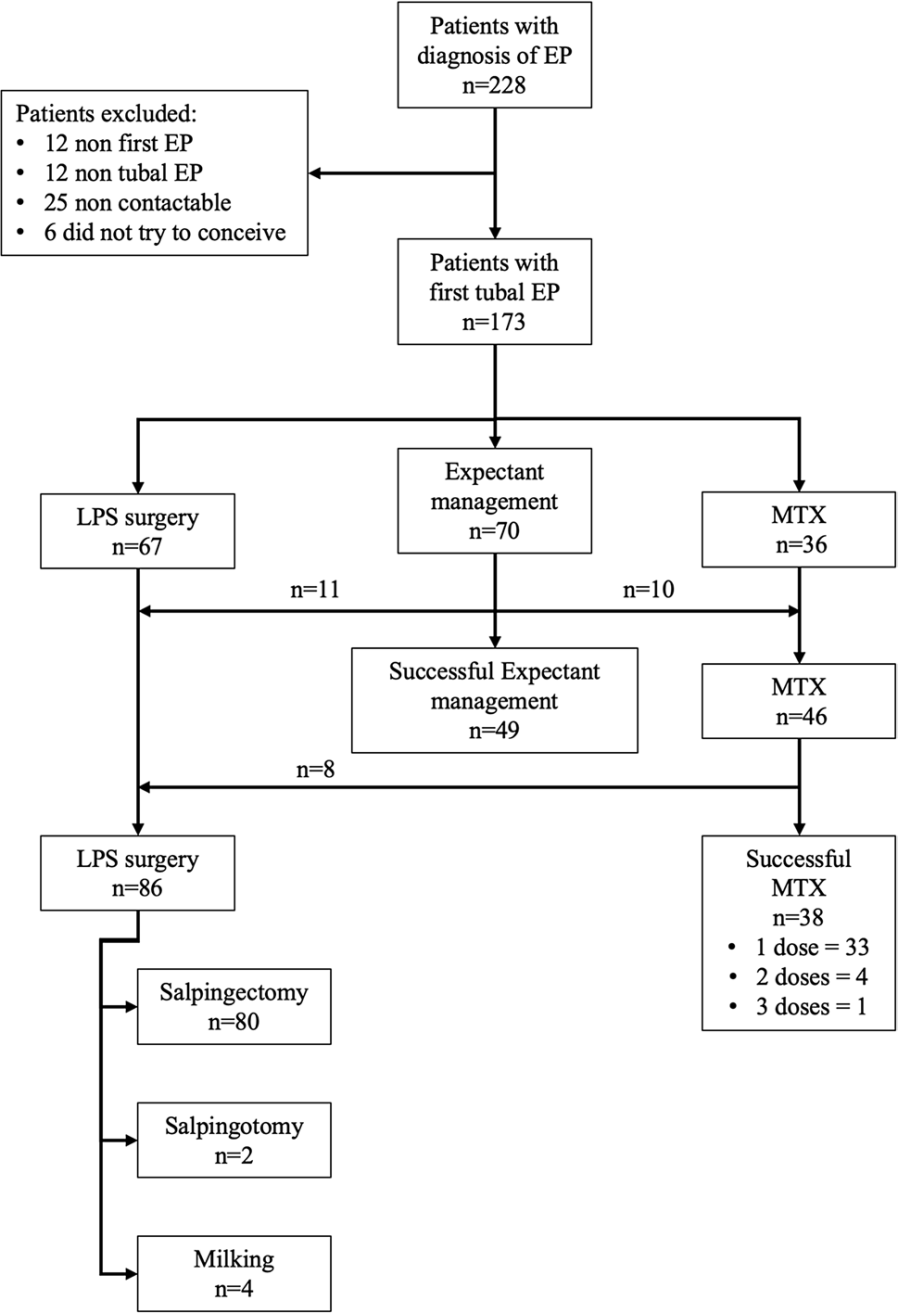
Flowchart of study population (*EP* ectopic pregnancy, *LPS* laparoscopy, *MTX* methotrexate)

One hundred seventy-nine out of 204 (87.7%) eligible patients responded to the phone call, and 173 out of 179 (96.6%) women tried to conceive after the index EP episode and agreed to participate. The interval between discharge from the hospital and follow-up ranged from 12 to 80 months. Seventy out of 173 (28.5%) patients with a diagnosis of tubal EP had initial expectant management, but only 49 (70%) did not receive any treatment following a “wait-and-see” approach. Ten (14.3%) women had plateau βhCG levels and they were subsequently managed with MTX, and another 11 women complained abdominal pain associated with increased levels of βhCG and had surgical management. Overall, 46 out of 173 (26.6%) received MTX, but in 8 (17.4%) of them, there was the failure of this therapy and were managed surgically. Finally, a total of 86 (49.9%) women had surgical management. The final success rates were 70% (49/70), 82.6% (38/46), and 100% (86/86) for expectant management, MTX, and surgery, respectively. Based on an intention to treat analysis, the success rate was 70% (49/70) for the expectant management, 83.3% (30/36) for the MTX, and 100% (67/67) for surgery. A flow-chart of patients with ectopic pregnancy was represented in Fig. 1. Patients’ characteristics are reported in Table 2 stratified per definitive treatment.

**Table 2:** Characteristics of the study population at the admission stratified per definitive treatment

	Expectant (*n* = 49)	MTX (*n* = 38)	Surgery (*n* = 86)	*p*-value
Demographic characteristic				
Age (years)	37.76 ± 5.68	36.29 ± 6.07	38.13 ± 5.26	0.233^1^
BMI (kg/m^2^)	24.09 ± 3.14	23.29 ± 5,04	22.28 ± 4.11	0.505^1^
Ethnic group				
Caucasian	30 (61.2%)	27 (71.1%)	51 (59.3%)	0.708^4^
East Europe	7 (14.3%)	6 (15.8%)	13 (15.1%)	
Africa	7 (14.3%)	3 (7.9%)	8 (9.3%)	
South-East Asia	3 (6.1%)	2 (5.3%)	11 (12.8%)	
South America	2 (4.1%)	0	3 (3.5%)	
Smoke (*n*)	3 (6.1%)	3 (8.6%)	12 (15.2%)	0.246^4^
Parity				
0	23 (46.9%)	14 (36.8%)	35 (40.6%)	0.242^3^
> 0 no LB	11 (22.4%)	9 (23.6%)	11 (12.7%)	
> 0 at least one LB	15 (30.6%)	15 (39.4%)	40 (46.5%)	
Clinical data of the day of recovery				
Gestational age				
(weeks)	5.52 ± 1.81^a^	6.34 ± 1.36^a,b^	6.32 ± 1.66^b^	0.037^2^
(days)	38 ± 12	44 ± 10	44 ± 11	
IUI	0	0	2 (2.3%)	0.359^4^
IVF	1 (2.0%)	2 (5.3%)	4 (4.7%)	0.693^4^
Additional US finding				
Blob or bagel sign	34 (69.4%)	33 (86.8%)	60 (69.8%)	0.088^3^
Hemoperitoneum	5 (10.2%)	2 (5.3%)	16 (18.6%)	
None	10 (20.4%)	3 (7.9%)	10 (11.6%)	
Mass size US (mm)	20.79 ± 11.75	19.36 ± 8.88	26.09 ± 15.27	0.098^2^
Hb (g/L)	127.10 ± 10.45^a,b^	130.01 ± 10.23^a^	121.54 ± 14.59^b^	0.011^2^
Leucocytes (*10^9^)	8.95 ± 2.64	8.30 ± 2.00	9.81 ± 3.82	0.134^2^
PLT (*10^9^)	252.90 ± 62.50	264.87 ± 58.41	256.59 ± 53.32	0.617^1^
ALT (U/L)	19.4 ± 7.12	18.85 ± 10.99	20 ± 6.96	0.349^2^
Creatinine (μmol/L)	59.9 ± 8.91	60.58 ± 8.45	62.33 ± 8.91	0.627^1^
βhCG (UI/L)	827.52 ± 1096.02^a^	913.58 ± 783.09^a^	6,165.18 ± 10,243.62^b^	< 0.001^2^
Precedent abdominal surgery				
No surgery	34 (69.4%)	29 (76.3%)	58 (67.4%)	0.147^4^
LPS	4 (8.2%)	4 (10.5%)	6 (7.0%)	
> 1 LPS	0	1 (2.6%)	1 (1.2%)	
LPT	7 (14.3%)	4 (10.5%)	19 (22.1%)	
> 1 LPT	4 (8.2%)	0	2 (2.3%)	

*MTX* methotrexate, *BMI* body mass index, *LB* live birth, *IUI* intrauterine insemination, *IVF* in vitro fertilization, *US* ultrasound, *Hb* hemoglobin, *PLT* platelet, *βhCG* beta-human chorionic gonadotropin, *LPS* laparoscopy, *LPT* laparotomy

^1^One-way independent ANOVA

^2^Kruskal–Wallis tests

^3^Pearson Chi-square test (0 cells with expected less than 5)

^4^Fisher exact test. Each subscript letter (a, b, c) denotes a subset of groups whose column do not differ significantly from each other at the 0.05 level after Bonferroni correction per multiple comparisons

About medical treatment, a single dose of MTX was enough for 33 (71.7%) patients, 4 (8.6%) patients required two doses, and one patient needed three doses. The eight women in the MTX group who failed initial management experienced increasing abdominal pain in the first weeks of follow-up after the first dose of MTX, and they required the surgical treatment. Nine (19.5%) women treated with MTX reported mild side effects, none needed additional doses, and the therapy was successful for all of them with the spontaneous improvement of all symptoms. Regarding the surgical technique, surgical treatments were salpingectomy for 80 (94.1%) women, salpingotomy for 2 (2.4%) and tubal milking for 4 (4.7%).

Women were evaluated about their fertility rate and reproductive outcomes after 12–80 months from the index EP. Ten out of 173 (5.8%) patients referred to assisted reproductive techniques for the attempt of conception after the index EP [six in vitro fertilization and embryo transfer (IVF-ET) and three intracytoplasmic sperm injection (ICSI), 1 intrauterine insemination (IUI)], and seven patients conceived; no significant different distribution regarding the modality of conception was observed among the three groups. Comparing the three treatments, we observed statistically significant differences in terms of cumulative incidence of intrauterine CP, LB, miscarriage, and time between the index EP and the first intrauterine CP (Table 3). In particular, expectant management was associated with the highest cumulative incidence of CP, LB, and the shortest time to achieve an intrauterine CP. However, surgery resulted to have the lowest proportion of women who experienced miscarriage. At the post-hoc pairwise analysis, only the expectant management reported statistically significant different reproductive outcomes compared to the surgical approach. Conversely, MTX treatment did not differ significantly from both the other two managements. No differences were observed regarding EP recurrences. The cumulative incidence of intrauterine CP after surgery was 37.5% (*n* = 30) for salpingectomy, 50% (*n* = 1) for salpingotomy and 75% (*n* = 3) for tubal milking, with a LB cumulative incidence of 27.5%, 0% and 75% respectively. No significant differences were observed according to the surgical methods in univariate analysis.

**Table 3:** Pregnancy outcome after treatment of ectopic pregnancy

	Expectant (*n* = 49)	MTX (*n* = 38)	Surgery (*n* = 86)	*p*
CP	32 (65.3%)^a^	21 (55.3%)^a,b^	34 (39.5%)^b^	0.012^1^
LB				0.035^2^
1	22 (44.8%)^a^	12 (31.5%)^a,b^	18 (20.9%)^b^	
> 1	3 (6.1%)^a^	1 (2.6%)^a^	7 (8.1%)^a^	
MIS				0.017^2^
1	10 (20.4%)^a^	8 (21.1%)^a^	3 (3.5%)^b^	
> 1	2 (4.1%)^a^	0^a^	2 (2.3%)^a^	
EP	1 (2%)	4 (10.5%)	7 (8.1%)	0.220^2^
EP_IUP time	7.92 ± 6.35^a^	13.11 ± 9.06^a,b^	12.87 ± 8.89^b^	0.041^3^

*CP* clinical pregnancy (at least one intrauterine pregnancy), *LB* live birth, *MIS* miscarriage, *EP* ectopic pregnancy, *IUP* intrauterine pregnancy, *EP_IUP time* Ectopic pregnancy to uterine pregnancy time (months), *MTX* methotrexate

^1^Pearson Chi-square test

^2^Fisher exact test

^3^Kruskal–Wallis tests. Each subscript letter (a, b, c) denotes a subset of groups whose column do not differ significantly from each other at the 0.05 level, after Bonferroni correction per multiple comparisons

The average size of the EP mass of women who had expectant management was 20.79 ± 11.75 mm, for women who received MTX was 19.36 ± 8.88 mm, and for women who underwent surgery was 26.09 ± 15.27 mm, and the difference was not statistically significant based on the conventional cut-off (*p* = 0.098; Table 2). However, being the EP mass diameter a possible confounder with the mandatory surgical approach in the case of a diameter bigger than 35 mm, we repeated the analysis restricting the study population to patients with an EP mass size of less than 25 mm (Table 4). The three treatment options reported a statistically significant different cumulative incidence in terms of intrauterine CP and LB. Conversely, miscarriages, recurrent EP, and time to intrauterine CP were comparable (Table 4). The post-hoc pairwise analysis showed a statistically significant difference in intrauterine CP and LB only between expectant and surgical management; in particular, expectant management showed a better reproductive outcome.

**Table 4:** Pregnancy outcome after treatment of ectopic pregnancy considering two cut-offs: ectopic pregnancy (EP) diameter < 25 mm and beta-hCG values < 1745 mUI/mL

	EP mass < 25 mm				βhCG ≤ 1745 mUI/mL			
	Expectant (*n* = 24)	MTX (*n* = 24)	Surgery (*n* = 31)	*p*	Expectant (*n* = 45)	MTX (*n* = 33)	Surgery (*n* = 26)	*p*
CP	18 (72%)^a^	12 (48%)^a,b^	7 (21.9%)^b^	0.001^1^	27 (61.4%)	17 (48.6%)	9 (33.3%)	0.071^1^
LB								
1	11 (45.8%)^a^	7 (29.2%)^a,b^	3 (9.7%)^b^	0.011^2^	17 (38.6%)	10 (30.3%)	5 (19.2%)	0.445^2^
> 1	3 (12.5%)^a^	1 (4.2%)^a^	2 (6.5%)^a^		3 (6.8%)	1 (3%)	2 (7.7%)	
MIS								
1	5 (20%)	5 (20%)	2 (6.3%)	0.139^2^	9 (20.5%)	8 (22.9%)	1 (3.7%)	0.135^2^
> 1	2 (8%)	0	0		2 (4.6%)	0	1 (3.7%)	
EP	1 (4%)	2 (8%)	4 (12.5%)	0.553^2^	1 (2.3%)	4 (11.4%)	2 (7.4%)	0.252^2^
EP_IUPtime	8.64 ± 7.51	11.54 ± 8.65	11.89 ± 5.54	0.479^3^	6.94 ± 5.78^a^	13.11 ± 9.06^a,b^	12.11 ± 4.01^b^	0.032^3^
EG	5.54 ± 1.67	6.4 ± 1.61	6.00 ± 1.33	0.207^3^	5.52 ± 1.81	6.18 ± 2.11	6.33 ± 1.36	0.145^3^

*CP* clinical pregnancy (at least one intrauterine pregnancy), *LB* live birth, *MIS* miscarriage, *EP* ectopic pregnancy, *IUP* intrauterine pregnancy, *EP_IUP time* ectopic pregnancy to uterine pregnancy time (months), *MTX* methotrexate, *βhCG* beta-human chorionic gonadotropin

^1^Pearson Chi-square test

^2^Fisher exact test

^3^Kruskal–Wallis tests. Each subscript letter (a, b, c) denotes a subset of groups whose column do not differ significantly from each other at the 0.05 level, after Bonferroni correction per multiple comparisons

In women with expectant management βhCG level was 827.52 ± 1096.02 UI/L, in patients who received MTX injections it was 913.58 ± 783.08 UI/L, and for women who underwent surgery, it was 6165.18 ± 10,243.62 UI/L (*p* < 0.001; Table 2). In the post-hoc pairwise analysis, expectant and MTX management were comparable; conversely, both reported βhCG values statistically significantly lower than the surgery group. Being the βhCG level at diagnosis (0 h) a possible confounder, we repeated the analysis stratifying women based on the βhCG cut-off of 1745 UI/L. In the groups with βhCG values lower than 1745 UI/L (Table 4), we found no statistically significant differences between the three groups, with the only exception for the length of time between the index EP and subsequent intrauterine CP. The expectant management showed the shortest length of time as compared to MTX and surgery, which was statistically significantly different at the pairwise analysis only between expectant and surgical management. For the βhCG level at 0 h more than 1745 UI/L, only one of the women treated with MTX had a subsequent CP, while the CP cumulative incidence after surgery was 37.9% and with expectant management was 100% (*p* = 0.017). Only the expectant management was statistically significantly different as compared to the other two approaches. For all the other outcomes the three groups were comparable.

## Discussion

In the past, EP has been one of the most important causes of maternal morbidity and mortality in the first trimester, accounting for 13% of the mortality rate [22], but in the last years, due to an earlier diagnosis, its impact has changed allowing a more conservative approach in several situations. In the case of tubal EP smaller than 35 mm and without embryos heartbeat, if the patient is clinically stable without signs or symptoms of tubal rupture or hemorrhage, three options are available (Table 1). Treatment options include expectant management, medical therapy with MTX, and surgery [23], and the common themes emerging during the discussion with women having clinically suspected EP are the concerns about the treatment effectiveness, the prognosis of future fertility, and the risk of recurrent EP.

Regarding the treatment efficacy, the data are available and comparable to our results, in which we observed 70% of cases successfully managed with the expectant approach, 82.6% of cases successfully treated with MTX, and 100% of success for surgery [24, 25]. Conversely, the available evidence about the fertility prospects after tubal EP pregnancy treatment is limited. The 2016 RCOG Green-top Guideline stated “there is no difference in the rate of fertility, the risk of future tubal ectopic pregnancy or tubal patency rates between the different management methods” based on low-quality evidence and expert opinion 8/17/2020 5:13:00 p.m.

Our results support this conclusion only partially. Expectant management was associated with the highest cumulative incidence in terms of intrauterine CP and LB, and to the shortest time interval between the index EP and the intrauterine CP as compared to MTX and surgery, although statistically significant differences were reported only compared to the surgical approach. Therefore, our results suggest that expectant management should be considered the treatment of choice when clinical conditions permit it. These results are further confirmed by the analysis limited to the population with an EP mass diameter lower than 25 mm. Conversely, in the stratified analysis based on the βhCG level, only a consistent trend of better reproductive outcomes in women underwent expectant management, without statistically significant differences, was observed; with the only exception for a statistically significant shorter time between the index EP and the subsequent intrauterine CP for the expectant management as compared to the surgical approach.

In general, our results showed a trend of better reproductive outcomes from the surgical approach to the expectant management through the MTX administration, which was confirmed in the stratified analyses for EP diameter and βhCG level. Nevertheless, the group that received MTX did not statistically significantly differ from both the expectant management and surgical approach, and the limited sample size of our study population does not allow to exclude a difference between the MTX administration and the other two approaches. However, for what concerns the comparison between MTX and surgery, this is consistent with the results of a meta-analysis that, comparing laparoscopy versus MTX in case of unruptured hemodynamically stable EP, showed that systemic MTX was more cost-effective, with less hospitalization, faster recovery, with no significant differences in subsequent spontaneous conception rate or recurrent ectopic pregnancy [26, 27]. Regarding the concern of leaving a damaged tube that can increase the risk of recurrence after surgery, no statistically significant differences were reported between the three therapeutic options, although a difference in cumulative proportion up to 8.5% was reported with lower values in the expectant management group as compared to MTX (10%) and surgery (8.1%), which were more comparable according to previous studies [28, 29].

About surgery, we did not find any significant difference in reproductive outcomes between the different surgical techniques (salpingectomy, salpingostomy and tubal milking), according to some previous studies [7, 30]. Nevertheless, our results are limited by the high proportion of patients underwent salpingectomy (94.1%), which limits the cases managed with other approaches and the related study power to show a difference. The high proportion of patients managed by salpingectomy was because it is the treatment of choice at our Institution; indeed, other studies clearly reported a lower rate of recurrence with no significant difference in future fertility after salpingectomy as compared to salpingotomy [7, 18, 19].

### Strengths and limitations

The provided evidence about the prognosis of future fertility in these women is limited, mainly because it is affected by confounding factors such as maternal age, previous infertility, history of pelvic/abdominal surgeries or inflammatory diseases, and tubal patency [11]. In our study, age, smoke habit, previous surgeries, BMI, and parity were comparable between the three groups of patients, allowing to exclude a possible effect of these confounders. Moreover, another confounder can be the absence of precise instructions regarding the choice of treatment, which depended on physician preference at the time of diagnosis, in particular in the case of intermediate situations [8, 10]. Instead, in our study, a pre-approved protocol adopted before the study period guided the choice of treatment option in all cases. Finally, the inclusion of only women who actively tried to conceive after the first tubal EP and the long follow-up (12–80 months) strength the study results and limit possible bias present in previous reports [30, 31].

Regardless of strengths, this study has some limitations, which need to be considered for appropriate interpretation of results. Although the investigation of the reproductive outcomes stratified per βhCG values and EP mass diameter allowed to reduce the effect of these possible confounders, the results must be read considering that for high βhCG values a comparison is difficult because only surgery was performed according to the protocol, as well as for the increase of the EP diameter. The fact that the chosen treatment was primarily guided by the βhCG value and EP mass diameter based on the protocol, which is intrinsically related to the characteristics of the EP, represents the main limitation of the present study. Indeed, we cannot completely exclude that the observed differences between treatments are related to the EP itself instead of the treatment. Nevertheless, assuming that the observed results are related to the EP instead of the treatment, which reflects the underlining characteristics of the EP, this study provides, in any case, evidence able to guide the counseling of patients. In this regard, we cannot confirm that these results are valid if the tubal EP is not managed following the reported protocol (Table 1). These limitations are mainly related to the retrospective study design with the non-randomization of patients to different treatments, which can introduce the possible above-mentioned confounders and limits statistical analysis. Other limitations are the limited sample size, which may explain the absent statistical significance in some observed differences, and the operator-dependence of the US measurement of the EP. Moreover, we cannot completely exclude false positive cases at US, with early intrauterine spontaneous miscarriages confused as EP, although we increased as much as possible the certainty about the diagnosis of tubal EP including in the population only patients with evidence of an EP mass at US.

## Conclusion

Tubal EP is often diagnosed in women who are trying to conceive; therefore, the prognosis of future fertility is one of the main concerns associated with this diagnosis. The results of the present study suggest a progressively better reproductive prognosis from the surgical approach to the expectant management, through the MTX administration, which was confirmed in the stratified analyses for EP diameter and βhCG level. Particularly, better reproductive outcomes are reported for expectant management as compared to the surgical approach. Even if the observed fertility outcomes should be more related to the EP mass diameter and the βhCG values rather than the adopted treatment, our study provide evidence able to guide the counseling for these patients, particularly if the expectant management is an option. If according to the protocol the expectant management is the treatment of choice, women should be informed that it is effective in more than two-thirds of patients, it is the less invasive option, and it is related to a better prognosis in term of future fertility, with the shortest time to achieve the next intrauterine pregnancy. Conversely, when expectant management is not applicable, medical treatment should be preferred, taking into account women’s preferences, and also because it has fewer anesthesia- and surgery-related risks [32]. However, given the unclear differences for subsequent fertility with surgery, the surgical treatment should be considered for women who desire to solve the problem as soon as possible, particularly in the presence of recurrent EP, and for patients whose compliance with immediate follow-up may be doubtful. When clinical presentation and protocol suggest that surgery is the safer and the preferred option, especially in cases of big EP mass with high βhCG values, surgery has to be adopted to prevent severe complications such as life-threatening bleedings.
